# The Physiology and Proteomics of Drought Tolerance in Maize: Early Stomatal Closure as a Cause of Lower Tolerance to Short-Term Dehydration?

**DOI:** 10.1371/journal.pone.0038017

**Published:** 2012-06-13

**Authors:** Monika Benešová, Dana Holá, Lukáš Fischer, Petr L. Jedelský, František Hnilička, Naďa Wilhelmová, Olga Rothová, Marie Kočová, Dagmar Procházková, Jana Honnerová, Lenka Fridrichová, Helena Hniličková

**Affiliations:** 1 Department of Genetics and Microbiology, Faculty of Science, Charles University in Prague, Prague, Czech Republic; 2 Department of Experimental Plant Biology, Faculty of Science, Charles University in Prague, Prague, Czech Republic; 3 Department of Cell Biology, Faculty of Science, Charles University in Prague, Prague, Czech Republic; 4 Department of Parasitology, Faculty of Science, Charles University in Prague, Prague, Czech Republic; 5 Laboratory of Mass Spectrometry, Faculty of Science, Charles University in Prague, Prague, Czech Republic; 6 Department of Botany and Plant Physiology, Faculty of Agrobiology, Food and Natural Resources, Czech University of Life Sciences Prague, Prague, Czech Republic; 7 Institute of Experimental Botany, Academy of Sciences of the Czech Republic, Prague, Czech Republic; Nanjing Agricultural University, China

## Abstract

Understanding the response of a crop to drought is the first step in the breeding of tolerant genotypes. In our study, two maize (*Zea mays* L.) genotypes with contrasting sensitivity to dehydration were subjected to moderate drought conditions. The subsequent analysis of their physiological parameters revealed a decreased stomatal conductance accompanied by a slighter decrease in the relative water content in the sensitive genotype. In contrast, the tolerant genotype maintained open stomata and active photosynthesis, even under dehydration conditions. Drought-induced changes in the leaf proteome were analyzed by two independent approaches, 2D gel electrophoresis and iTRAQ analysis, which provided compatible but only partially overlapping results. Drought caused the up-regulation of protective and stress-related proteins (mainly chaperones and dehydrins) in both genotypes. The differences in the levels of various detoxification proteins corresponded well with the observed changes in the activities of antioxidant enzymes. The number and levels of up-regulated protective proteins were generally lower in the sensitive genotype, implying a reduced level of proteosynthesis, which was also indicated by specific changes in the components of the translation machinery. Based on these results, we propose that the hypersensitive early stomatal closure in the sensitive genotype leads to the inhibition of photosynthesis and, subsequently, to a less efficient synthesis of the protective/detoxification proteins that are associated with drought tolerance.

## Introduction

Drought is likely the most important environmental factor that adversely affects plant growth and development. Effects of drought on plants have been studied for a long time and changes induced by insufficient water supply have been examined from the whole plant/plant population level to biochemical and molecular level [1], [2]. The primary and the most rapidly developing symptom of water stress in plants is a cessation of cell expansion caused by a decrease of turgor. This reduces plant water use but negatively affects growth and development together with the reduction or suppression of cell division which is, however, much less sensitive to water deficit compared to cell expansion. Decrease of transpiration caused by partial or complete stomatal closure is associated with changes in both leaf water status and soil moisture content, the latter being mediated predominantly through signalling molecules produced by dehydrating roots, particularly the abscisic acid (ABA). A dependence of stomatal behaviour on air humidity and hydraulic conductivity of xylem was also found [1], [3]. The sensitivity of stomata to ABA can be regulated by additional factors like xylem sap pH, plant nutritional status, *etc*. The complex interplay between ABA and other growth regulators (particularly cytokinins and ethylene) in the induction of stomatal closure is far from being fully understood despite recent progress in this area [1], [3]–[8].

The closure of stomata naturally affects more processes than just transpiration: the limitation of CO_2_ uptake by leaves is closely linked to the stomatal control of water loss. The reduction in net carbon assimilation/photosynthetic rate (P_N_) and the decrease of intercellular CO_2_ concentration (c_i_) are thus usually regarded as another early symptoms of water stress. In the initial stages of water deficit, the reductive effect of stomatal closure on transpiration rate is greater than the effect on CO_2_ assimilation, but with further development of water deficit, both processes are often dramatically reduced. Actual contribution of the decrease in stomatal conductance (g_s_) and c_i_ to drought-induced limitation of photosynthesis has been much discussed during past decades (see [9]–[11] for recent reviews). Now, a majority of scientists working in this area of research accepts the „stomatal control” model. This model proposes that stomatal closure and decrease of g_s_ are the primary causes of the reduction of P_N_ under mild drought conditions. As to c_i_, the evaluation of the role of its changes for photosynthetic limitation is rather difficult, not only due to the existence of stomatal patchiness, but because of the important role of mesophyll conductance for the determination of CO_2_ concentration in chloroplasts of drought-stressed plants [2], [4], [10]–[15].

Biochemical limitations of photosynthetic carbon fixation (*i.e.* the inadequate regeneration of RuBP, the inhibition of ribulose-1,5-bisphosphate carboxylase/oxygenase (Rubisco) activity together with activities of other enzymes of photosynthetic carbon reduction cycle, as well as the inhibition of Rubisco activase caused by the reduction in ATP content) play an important role mostly under conditions of prolonged or more severe drought, though some of these changes were observed even in the early stages of drought stress [4], [15]–[19]. Moreover, there can be a differential inhibition of the C3 and C4 cycles enzymes [20]. Primary photosynthetic processes are rather resilient to water deficit and decrease of electron transport efficiency occurs usually only secondarily, caused by the imbalance between the generation of NADPH and its utilization in photosynthetic carbon reduction cycle (and, consequently, the imbalance between light capture and utilization, resulting in the photoinhibition). Severe drought can also lead to the increased generation of reactive oxygen species leading to photooxidation and degradation of photosynthetic membrane proteins and associated pigments and lipids, and disorganization of thylakoid membranes [16], [18], [21], [22].

Inhibition of photosynthetic metabolism results in the diminished amount of photosynthetic assimilates available for sucrose and starch synthesis. Moreover, the activity of sucrose phosphate synthase is also greatly reduced by water deficit and the ratio of starch/sucrose alters [19]. Sucrose, glucose and fructose are important components of drought-signaling pathways [2], [9]. The carbohydrate status in drought-stressed plants depends not only on the efficiency of photosynthetic carbon reduction cycle and sucrose/starch synthesis, but it is linked to the processes of osmotic adjustment as well [6], [23], [24]. Besides carbohydrates, other classes of osmolytes accumulate in cells of plants exposed to water deficiency, such as proline, glycine betaine, putrescine, γ-aminobutyric acid *etc*. Some of them also fulfill a stabilizing and protective role for cellular membranes and enzymes as they can interact with hydrophobic residues of proteins and reduce the rate of protein unfolding [1], [6], [24], [25]. Proteins belonging to the LEA (late embryogenesis abundant) family (including dehydrins) or small heat-shock proteins might act in a similar manner [23], [24], [26]. These proteins were among the first to be identified using cDNA library techniques that permitted differential screening for drought-induced genes [26].

Considerable advances in high-throughput methods of plant molecular and cell biology have enabled scientists to study the molecular events involved in plant response to drought in great detail and on a global scale. Various transcriptomic analyses have facilitated the large-scale dissection of the dehydration-induced changes in gene expression and have revealed several categories of genes that are differentially regulated in response to dehydration [27]–[33]. However, the molecular analysis of plant response to drought stress cannot be limited to the transcriptional level. The changes that occur in the cells of plants that are subjected to water deficiency ultimately depend on the interactions between and the modifications of a large number of proteins that participate in various metabolic, signaling, biosynthetic and degradation pathways and other important cellular processes. The quantities and functions of these proteins are regulated not only by the amounts of their mRNAs but also at the translational and post-translational levels and various discrepancies have been found between the amounts of transcripts and their respective proteins in drought-stressed plants [34]–[36]. The analysis of the plant proteome thus offers several advantages over transcriptomic methods for the large-scale study of the molecular changes associated with the drought stress response. Proteomics has already been used to evaluate drought-responsive proteins in the leaves of important crop species, such as rice [37]–[42], maize [43]–[48], wheat [36], [49], cotton [50], peanut [35], amaranth [51], alfalfa [52], sugar beet [53] and sunflower [54]. The simplest studies focused only on the dehydration-induced qualitative and quantitative changes of proteins. Several authors also compared the responses of tolerant and sensitive genotypes of a single species to drought conditions [35], [37], [39], [40], [43]–[46], [50], [55]–[59]. Such analyses can be very useful in revealing proteins that are directly involved in the mechanisms underlying plant tolerance to drought. These proteins can then serve as molecular markers in marker-assisted selection and breeding programs or in transgenic approaches to improving plant drought tolerance [40], [60]–[62].

To investigate the mechanism of the plant stress response, it is convenient to use a combination of biochemical and physiological measurements of stress response-relevant parameters and to monitor the qualitative and quantitative changes in the composition of proteins, which represent the executive component of the protective response. The care should be also taken to ascertain that the experimental conditions simulate water deficiency scenarios that the respective plant species is probable to encounter in the nature. Drought stress can be either mild/moderate (of a relatively short duration, with the possibility of periodical re-watering of soil) or severe drought stress that can be terminal (occuring in very dry environments with long periods of water deficiency; [11], [63]). Maize, one of the most important crop species, is known to be susceptible to even mild or moderate drought particularly at the heading stage; however, unfavourable soil water conditions at the beginning of plant growth may also dramatically limit the biomass production and the photosynthetic ability of leaves and thus indirectly negatively affect the formation of reproductive organs and yield parameters [64]. The presented study attempts to enhance our knowledge of maize responses to mild water deficiency at the early developmental stages in two maize genotypes that were chosen based on their different sensitivity to this abiotic stressor. To uncover the possible basis for drought tolerance we examined drought-induced changes that occured at both the physiological and the proteome level, which was analyzed by a combination of two techniques, 2DGE (two-dimensional gel electrophoresis) and iTRAQ (isobaric tag for relative and absolute quantitation).

## Results

### Analysis of Plant Morphology, Water Status, Leaf Gas Exchange Parameters and Antioxidant Enzymes Activities

**Figure 1:**
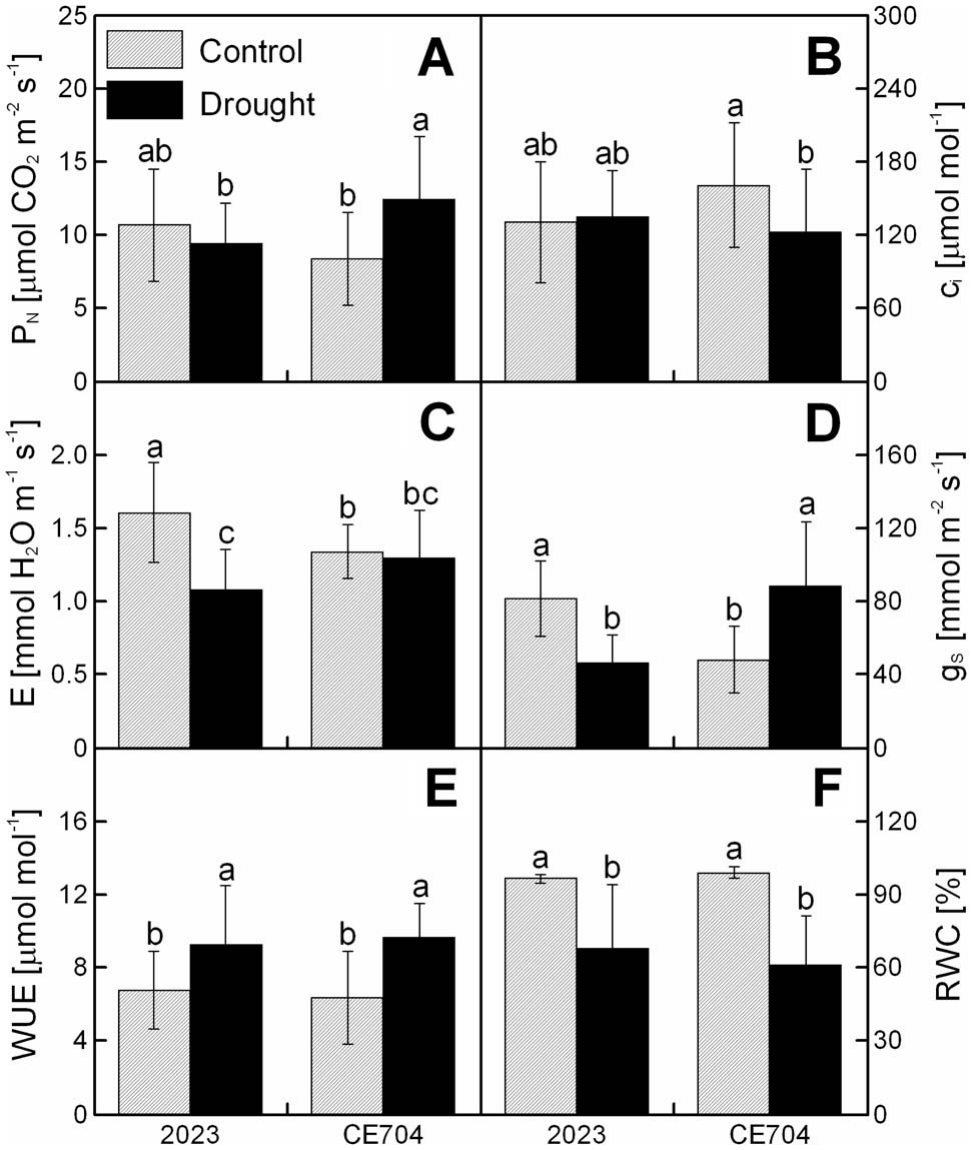
The gas exchange and water use characteristics of the leaves of drought-stressed maize genotypes. The net photosynthetic rate (P_N_) (***A***), intercellular CO_2_ concentration (c_i_) (***B***), net transpiration rate (E) (***C***), stomatal conductance (g_S_) (***D***), water use efficiency (WUE) (***E***) and relative water content (RWC) (***F***) in the leaves of two maize genotypes (2023 and CE704) that were subjected to 6 days of drought (solid bars) or normally watered (hatched bars). The means ± SD (n  = 18) are shown. The letters *a-c* denote the statistical significance (as determined by the Tukey-Kramer test) of the differences between genotypes/water treatments (only those marked with different letters differ significantly at p≤0.05).

**Figure 2:**
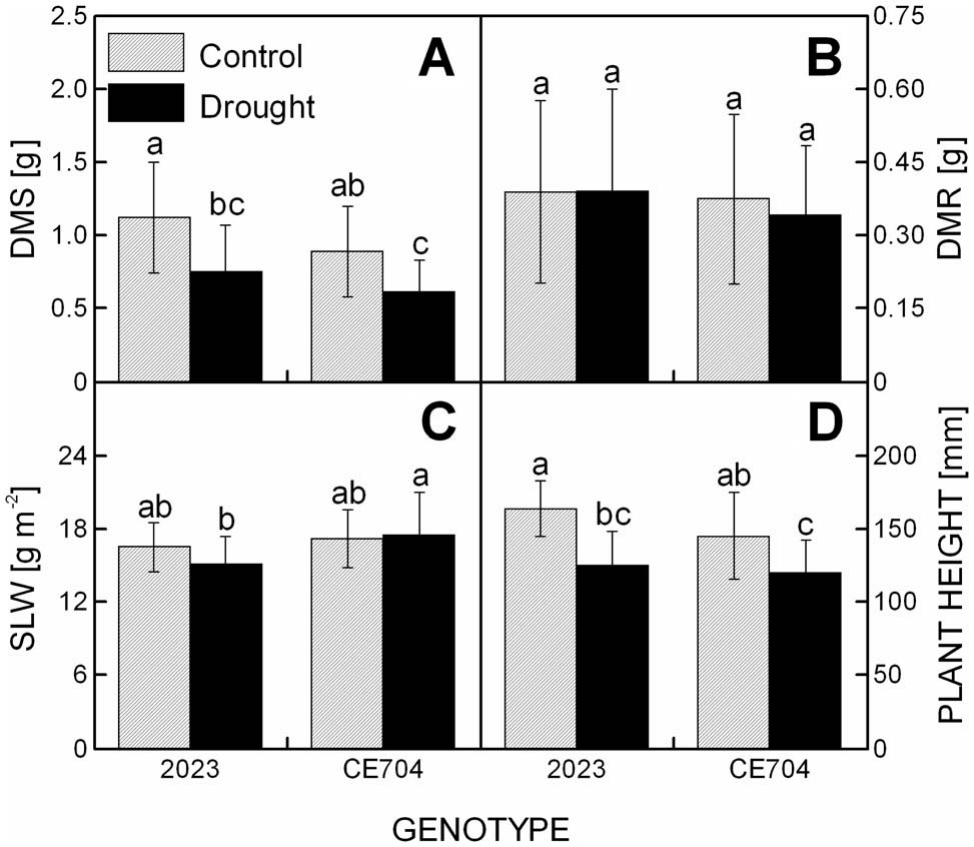
The morphology and biomass characteristics of drought-stressed maize genotypes. The dry mass of the shoot (DMS) (***A***), dry mass of the roots (DMR) (***B***), specific weight of the 4^th^ leaf (SLW) (***C***) and plant height (***D***) of two maize genotypes (2023 and CE704) subjected to 6 days of drought (solid bars) or normally watered (hatched bars). The means ± SD (n  = 20) are shown. The letters *a-c* denote the statistical significance (as determined by the Tukey-Kramer test) of the differences between genotypes/water treatments (only those marked with different letters differ significantly at p≤0.05).

Control plants of the CE704 genotype were characterized by significantly lower dry mass of the shoot to dry mass of the roots (DMS/DMR ratio) (mean±SD  = 2.56±0.57 in CE704 and 3.17±0.93 in 2023), g_S_ (Figure 1*D*) and E (Figure 1*C*) compared with the 2023 genotype. A 6-day treatment without watering resulted in a mild drought stress that was characterized by a statistically significant decline in the relative water content (RWC) in both examined genotypes (Figure 1*F*). CE704 was characterized by a slightly more pronounced decrease in the RWC (to approx. 61% of the control values), compared with 2023 (70% of control). The plants that were subjected to drought stress were also characterized by lower height (Figure 2*D*) and DMS (Figure 2*A*), respectively, compared with the control plants; the decrease in both of these parameters was slightly more pronounced in the 2023 genotype (77% and 67%) compared with CE704 (82% and 69%). The DMR did not change significantly with the drought treatment (Figure 2*B*) nor did the specific leaf weight (SLW), although the latter showed a slight decrease in the 2023 genotype (Figure 2*C*).

A significant increase in P_N_ (149% of the control) was observed in the CE704 plants that were subjected to dehydration but not in the 2023 plants (Figure 1*A*). The values of E did not change with the drought treatment in CE704 and were significantly decreased (67% of control) in 2023 (Figure 1*C*), whereas the reverse was true for c_i_ (Figure 1*B*). With regard to g_S_, the values of this parameter in the leaves of 2023 plants were significantly decreased (57% of the control), whereas a statistically significant increase (185% of the control) in this parameter was observed in CE704 (Figure 1*D*). An increase in the water use efficiency (WUE) due to drought stress was observed in both genotypes (136% of the control in 2023 and 151% of the control in CE704) (Figure 1*E*).

**Figure 3:**
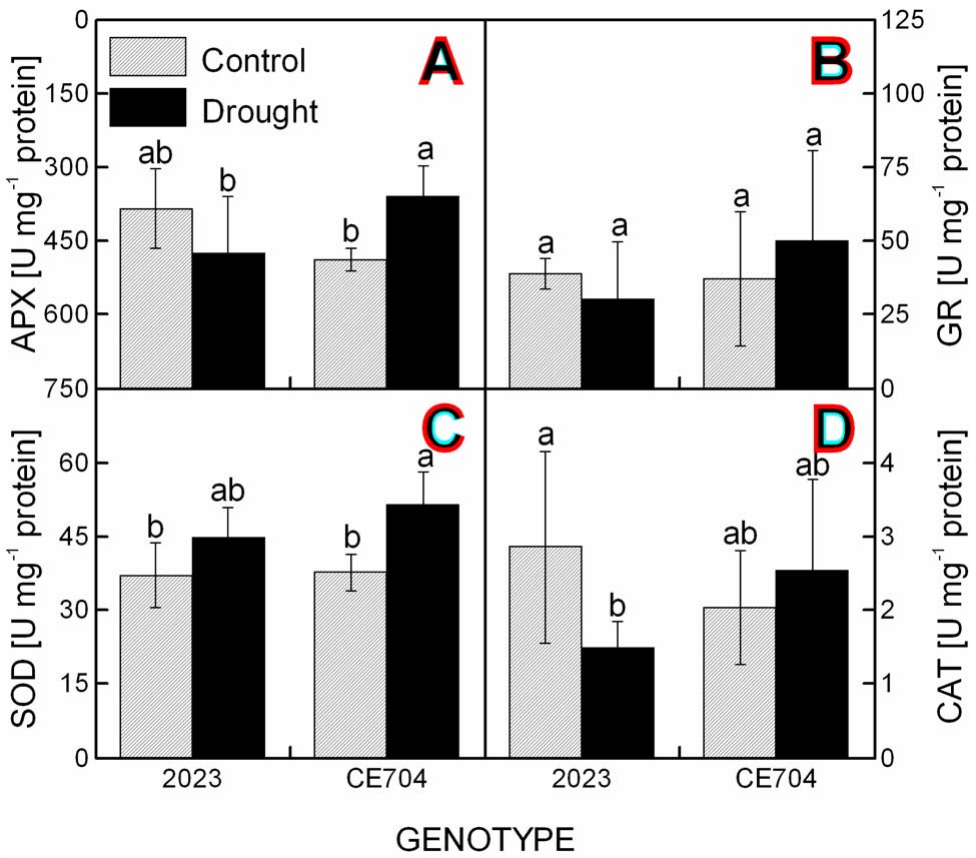
The activities of antioxidant enzymes in the leaves of drought-stressed maize genotypes. The activities of ascorbate peroxidase (APX) (***A***), glutathione reductase (GR) (***B***), superoxide dismutase (SOD) (***C***) and catalase (CAT) (***D***) in the leaves of two maize genotypes (2023 and CE704) subjected to 6 days of drought (solid bars) or normally watered (hatched bars). The means ± SD (n  = 8) are shown. The letters *a-b* denote the statistical significance (as determined by the Tukey-Kramer test) of the differences between genotypes/water treatments (only those marked with different letters differ significantly at p≤0.05).

The activities of the antioxidant enzymes in the leaves of the control plants did not differ significantly between the genotypes, although 2023 showed slightly higher ascorbate peroxidase (APX) and catalase (CAT) activities compared with CE704 (Figure 3). The drought conditions led to a significant increase in the APX (Figure 3*A*) and superoxide dismutase (SOD) (Figure 3*C*) activities in the CE704 genotype (to 149% and 137% of the control, respectively) and to a similar, although non-significant, increase in the activities of glutathione reductase (GR) and CAT (to 135% and 125% of control, respectively). In contrast, the activities of antioxidant enzymes in 2023 either decreased (to 52%, 78% and 75% of the control for CAT, APX and GR, respectively; Figure 3*A, 3B, 3D*) or showed only a non-significant increase (120% of the control for SOD; Figure 3*C*).

#### Analysis of the leaf proteome

**Figure 4:**
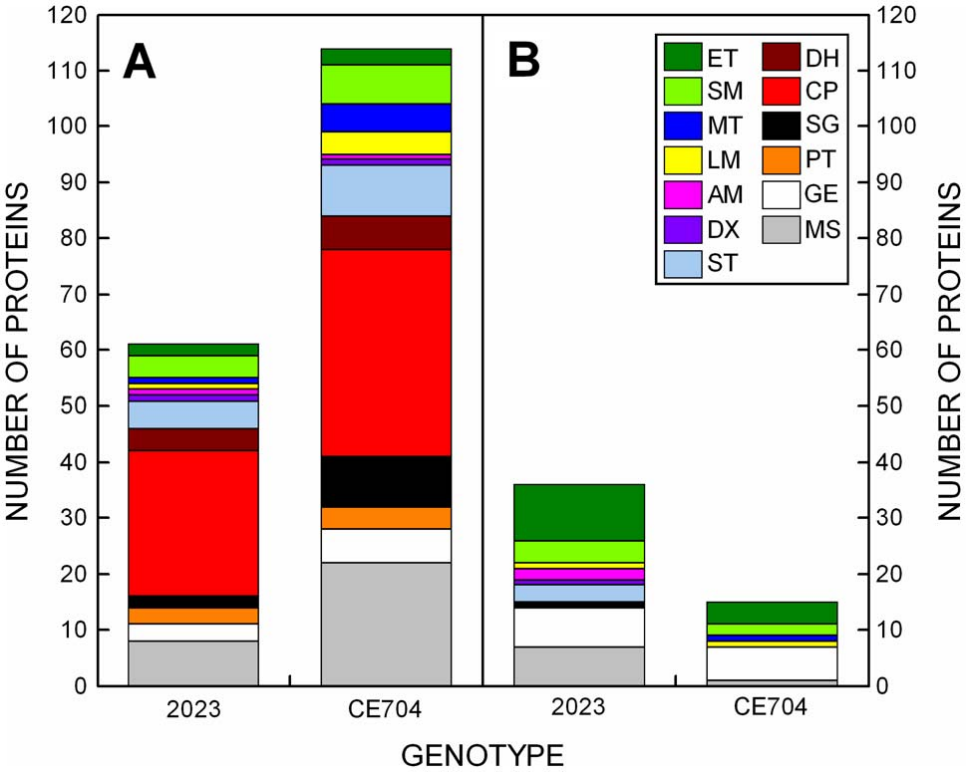
The functional classification of differentially expressed drought-related proteins from maize leaves. The number of proteins identified by the iTRAQ method in two maize genotypes (2023 and CE704) with up-regulated (***A***) or down-regulated (***B***) levels is shown; only those proteins whose levels changed due to drought in at least one genotype by at least twofold were included. ET: proteins of the photosynthetic electron-transport chain and chlorophyll synthesis; SM: proteins participating in photosynthetic carbon fixation and saccharide metabolism; MT: membrane proteins participating in transport; LM: proteins participating in lipid metabolism; AM: proteins participating in amino acid metabolism; DX: detoxification proteins; ST: stress proteins; DH: dehydrins; CP: chaperones; SG: proteins involved in cell signaling; PT: proteases and their inhibitors; GE: proteins participating in gene expression and its regulation; MS: miscellaneous proteins.

The proteomic changes induced during the drought period were analyzed by two independent approaches: the comparison of 2DGE and the iTRAQ analysis. The iTRAQ analysis revealed 1,244 unique peptides, out of which 326 unique peptides were identified using the NCBI protein database and 1,164 unique peptides using the NCBI EST database (245 peptides were present in both lists; Table S1). To identify the drought stress-related proteins, the results of the iTRAQ analysis were primarily expressed as three different ratios. The responses of the individual genotypes to stress were evaluated using the S_2023_/C_2023_ and S_CE704_/C_CE704_ ratios, *i.e.,* stressed *vs.* control plants of 2023 or CE704, respectively; for the proteins whose levels *decreased* in the stressed plants compared with the control, these ratios were expressed as –1/(S_2023_/C_2023_) or –1/(S_CE704_/C_CE704_). The third, derived ratio (S_CE704_/C_CE704_)/(S_2023_/C_2023_) (or −1/[(S_CE704_/C_CE704_)/(S_2023_/C_2023_)] for the down-regulated proteins) reflected the different responses of the genotypes to drought. Our attention was focused only on those peptides whose levels changed due to drought stress in at least one genotype by at least twofold, as inferred from the first two ratios. Additionally, the peptides whose levels changed differentially in the two genotypes (by at least twofold; inferred from the third ratio) were investigated. The total number of identified proteins fulfilling these criteria was 220. These proteins were classified into 13 groups based on their functions (Figure 4). In addition to proteins with various or unknown functions, which were assigned to the Miscellaneous category (21% of the total number of differentially expressed proteins), the most-represented group of proteins was comprised of chaperones (18%), whose concentration increased after drought stress in both genotypes. The energetic metabolism category (20%), consisting of the proteins associated with primary (10%) and secondary photosynthetic processes and saccharide metabolism (10%), was another significantly represented group. Proteins participating in gene expression and its regulation constituted an additional 12% of the total number of differentially expressed proteins. The other categories could be characterized as minor (each represented by less than 7% of the differentially expressed proteins).

**Figure 5:**
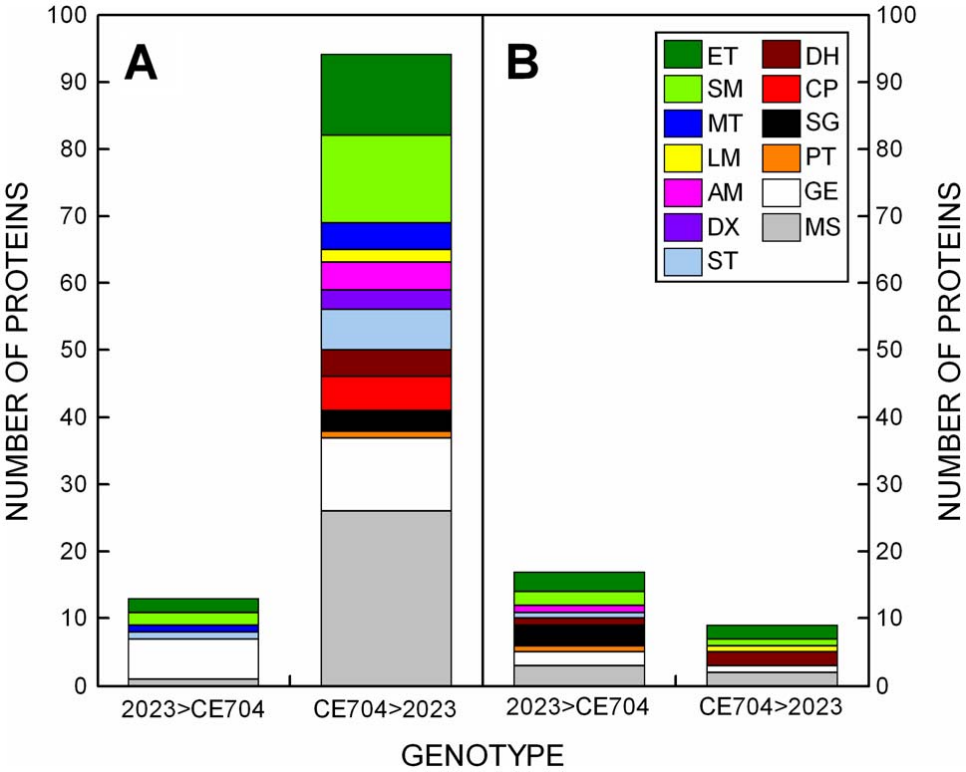
The functional classification of differentially expressed proteins from maize leaves with genotype-dependent contrasting responses to drought. The number of proteins identified by the iTRAQ method that were up-regulated in one genotype and down-regulated in the other genotype or *vice versa* is shown in panel (***A***); the number of proteins belonging to this category with different levels in control plants of both genotypes is shown in panel (***B***). Only proteins whose levels changed differentially in the two genotypes by at least twofold were included. 2023> CE704: up-regulation of protein levels in the 2023 genotype and down-regulation in the CE704 genotype (***A***) or higher level in control plants of the CE704 genotype compared with the 2023 genotype (***B***); CE704>2023: up-regulation of protein levels in the CE704 genotype and down-regulation in the 2023 genotype (***A***) or higher level in control plants of the 2023 genotype compared with the CE704 genotype (***B***); ET: proteins of the photosynthetic electron-transport chain and chlorophyll synthesis; SM: proteins participating in photosynthetic carbon fixation and saccharide metabolism; MT: membrane proteins participating in transport; LM: proteins participating in lipid metabolism; AM: proteins participating in amino acid metabolism; DX: detoxification proteins; ST: stress proteins; DH: dehydrins; CP: chaperones; SG: proteins involved in cell signaling; PT: proteases and their inhibitors; GE: proteins participating in gene expression and its regulation; MS: miscellaneous proteins.

The majority of the proteins identified by the iTRAQ responded to drought stress similarly in both genotypes; however, 106 out of 220 differentially expressed and identified proteins were up-regulated in one genotype and down-regulated in the other genotype or *vice versa*; CE704 was usually characterized by the up-regulation of these proteins and 2023 by the down-regulation of their levels (Figure 5*A*). Among these, 26 proteins showed significant differences between both genotypes even in control plants (Figure 5*B*). The total number of proteins up-regulated above the twofold limit was much higher in CE704 (114 proteins) compared with 2023 (61 proteins); for down-regulated proteins, the situation was reversed (15 proteins in CE704, 36 proteins in 2023) (Figure 4). The most extreme responses to drought stress in the leaf proteome are presented in Table 1, which shows 5 proteins whose levels were the most strongly up- and down-regulated in the two genotypes examined. With one exception, the same proteins showed the highest accumulation during dehydration in both genotypes, with the majority of these proteins belonging to the category of chaperones (Figure 4*A*, Table 1). In contrast, the most strongly down-regulated proteins differed markedly between the genotypes and were primarily involved in the regulation of gene expression, photosynthesis and saccharide metabolism (Figure 4*B*, Table 1). The proteins that most strongly differed between the CE704 and 2023 genotypes in the dehydration-induced up−/down-regulation of their levels are listed in Table 2.

**Table 1:** Five most up-regulated and down-regulated proteins in drought-stressed maize plants of 2023 and CE704 genotypes, as revealed by the iTRAQ analysis.

Protein	CE704	2023	Matching sequence (EST/protein)	Closest homolog with known function	Species	Functional category
**Ranked according to the CE704 genotype**						
Dehydrin RAB-17	30.1	15.0	gi|149074542	ref|NM_001111949.1	***ZM***	Dehydrins
Dehydrin RAB-17	16.2	6.9	gi|239236	gi|239236	*ZM*	Dehydrins
Hypothetical protein	14.2	4.4	gi|148953111	gb|EU953517.1	*ZM*	Miscellaneous
Heat shock protein 16.9 kDa (AC-type class I)	13.6	7.7	gi|92088239	ref|NM_001157311.1	*ZM*	Chaperons
Hypothetical protein	13.0	6.1	gi|23928441	gi|23928441	*ZM*	Miscellaneous
Ribonucleoprotein A	–4.6	–2.1	gi|149065598	ref|NM_001158256.1	*ZM*	Gene expr. + regulation
AT-hook protein 1	–3.2	–1.6	gi|148955887	gb|EU959419.1	*ZM*	Gene expr. + regulation
Sugar carrier protein C	–3.0	1.5	gi|148966293	ref|NM_001154535.1	*ZM*	Membrane + transport
WD-repeat protein	–3.0	1.5	gi|89247710	gb|EU958180.1	*ZM*	Gene expr. + regulation
Nicotinate phosphoribosyltransferase-like protein	–3.0	1.5	gi|101398157	ref|NM_001159021.1	*ZM*	Miscellaneous
**Ranked according to the 2023 genotype**						
Dehydrin RAB17	30.1	15.0	gi|149074542	ref|NM_001111949.1	*ZM*	Dehydrins
Heat shock protein 16.9 kDa (AC-type class I)	13. 6	7.7	gi|92088239	ref|NM_001157311.1	*ZM*	Chaperons
Dehydrin RAB-17	16.2	6.9	gi|239236	gi|239236	*ZM*	Dehydrins
Hypothetical protein	13.0	6.1	gi|23928441	gi|23928441	*ZM*	Miscellaneous
Heat shock protein 17.4 kDa (AC-type class I)	8.3	6.0	gi|149109747	gb|EU962980.1	*ZM*	Chaperons
Elongation factor 1-delta (eEF1D)	1.9	–5.2	gi|116814898	ref|NM_001155791.1	*ZM*	Gene expr. + regulation
Nucleoside-triphosphatase (disease resistance gene analog PIC15)	1.2	–3.4	gi|3982622	gi|3982622	*ZM*	Stress proteins
Ferredoxin	1.2	–3.2	gi|48374987	gi|48374987	*ZM*	Photosynthetic ETC
Ribonucleoprotein A	–2.8	–3.1	gi|149083997	gb|EU972036.1	*ZM*	Gene expr. + regulation
Phosphatase PHOSPHO1 (phosphoethanolamine/phosphocholine phosphatase)	–2.3	–3.0	gi|149104232	gb|EU953126.1	*ZM*	Miscellaneous

The number in the column “CE704”, resp. “2023”, represents the n-fold increase or decrease in the protein content after 6 days of drought, derived from the ratio S_CE704_/C_CE704_ (resp. S_2023_/C_2023_) in case of the increased protein content and from the formula: –1/(S_CE704_/C_CE704_) (resp. –1/[S_2023_/C_2023_]) in case of the decreased protein content. ETC  =  electron transport chain; ZM  =  *Zea mays* L.

**Table 2:** Five proteins with the strongest contrast in the response to drought between 2023 and CE704 maize genotypes.

Protein	CE704	2023	Contrast	Matching sequence (EST/protein)	Closest homolog with known function	Species	Functional category
**Proteins with higher up-regulation in the CE704 genotype/higher down-regulation in the 2023 genotype**							
Elongation factor 1-delta (eEF1D)	1.90	–5.18	9.84	gi|116814898	ref|NM_001155791.1	*ZM*	Gene expr. + regulation
Hydrolase (alpha/beta fold family)	3.82	–2.30	8.80	gi|149074075	ref|NM_119816.4	*AT*	Miscellaneous
KDE-like protein (cylicin 1)	3.33	–2.01	6.68	gi|113703231	ref|NM_001155938.1	*ZM*	Miscellaneous
Xylanase inhibitor (TAXI-IV)	3.52	–1.76	6.20	gi|149112160	gb|EU976729.1	*ZM*	Saccharide metabolism
PsaK (photosystem I reaction center subunit)	2.05	–2.45	5.03	gi|149205590	gb|EU967431.1	*ZM*	Photosynthetic ETC
**Proteins with higher up-regulation in the 2023 genotype/higher down-regulation in the CE704 genotype**							
Transaldolase 2	–1.7	1.7	–2.9	gi|149099949	ref|NM_001157202.1	*ZM*	Saccharide metabolism
Ribosomal protein S18	–1.4	2.2	–3.0	gi|166528395/gi|11467213	gi|11467213	*ZM*	Gene expr. + regulation
Nicotinate phosphoribosyltransferase-like protein	–3.0	1.5	–4.5	gi|101398157	ref|NM_001159021.1	***ZM***	Miscellaneous
WD-repeat protein	–3.0	1.5	–4.5	gi|89247710	gb|EU958180.1	*ZM*	Gene expr. + regulation
Sugar carrier protein C	–3.0	1.5	–4.5	gi|148966293	ref|NM_001154535.1	*ZM*	Membrane + transport

The number in the column “CE704”, resp. “2023”, represents the n-fold increase or decrease in the protein content after 6 days of drought, derived from the ratio S_CE704_/C_CE704_ (resp. S_2023_/C_2023_) in case of the increased protein content and from the formula: –1/(S_CE704_/C_CE704_) (resp. –1/[S_2023_/C_2023_]) in case of the decreased protein content. The number in the column “Contrast” represents the difference between genotypes according to the ratio (S_CE704_/C_CE704_)/(S_2023_/C_2023_) in case of the higher protein up-regulation in the CE704 genotype/higher protein down-regulation in the 2023 genotype (upper part of the table). For the opposite situation (lower part of the table), the formula: –1/([S_CE704_/C_CE704_]/[S_2023_/C_2023_]) was used. AT  =  *Arabidopsis thaliana* (L.) Heynh.; ETC  =  electron transport chain; ZM  =  *Zea mays* L.

**Figure 6:**
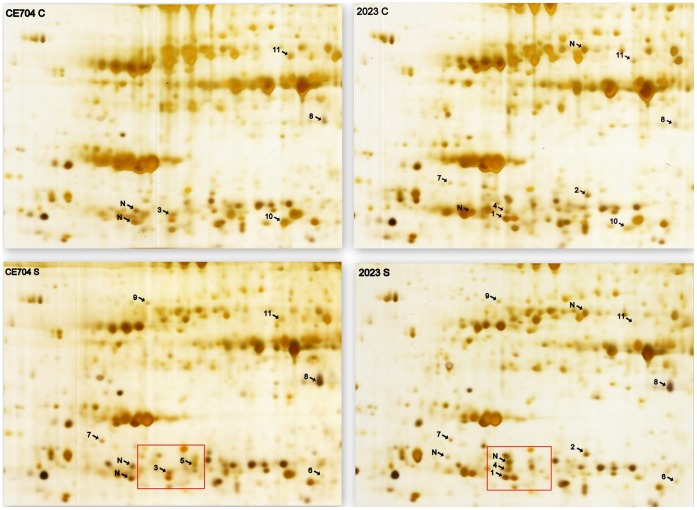
The 2D gels showing the leaf proteomes of drought-stressed and control plants of two maize genotypes. S: drought-stressed; C: control; 2023: sensitive genotype; CE704: tolerant genotype. Only selected regions of the gels are shown; the frames mark the differences in the representation of two isoforms of the heat-shock protein HSP26 (spots nos. 4 and 5) in the drought-stressed plants of both genotypes. The protein spots that are differentially represented between genotypes and water treatments are marked by arrows and the respective numbers (1–11; N … unidentified protein) refer to the notation used in Table 3.

The 2DGE analysis yielded approximately 300 spots that were clearly visible in each gel (Figure 6); among them, 17 spots showed strong differences in their presence/position or their intensity either between genotypes or between the plants subjected to the control or drought conditions. The MALDI-TOF MS/MS analysis and database searches identified 11 of these proteins (Table 3).

**Table 3:** The differences in leaf proteins observed either between the genotypes or between control (C) and drought-stressed (S) plants of 2023 and CE704 maize genotypes, as evaluated by the 2D-electrophoresis method.

Spot number	Protein	2023 C	2023 S	CE704 C	CE704 S	Matching sequence (EST/protein/GSS)	Closest homolog with known function	Species	Functional category
1	Chaperonin 20 (21 kDa)	+	+	–	–	gi|91049630	gb|AF233745.1|AF233745	***LE***	Chaperons
2	Glutathione S-transferase GST 27	+	++	–	–	gi|162462462	gi|162462462	*ZM*	Detoxification
3	Triosephosphate isomerase	–	–	+	++	gi|91056160	gb|EU959275.1	*ZM*	Saccharide metabolism
4	Heat shock protein 26	+/−	+	–	–	gi|162461165	gi|162461165	*ZM*	Chaperons
5	Heat shock protein 26	–	–	–	+	gi|162461165	gi|162461165	*ZM*	Chaperons
6	Glutathione transferase 5	–	+	–	+	gi|162458026	gi|162458026	*ZM*	Detoxification
7	IN2-1 protein	+/−	+	–	+	gi|148938867	ref|NM_001155003.1	*ZM*	Miscellaneous
8	Stress-responsive protein	+	++	+	++	gi|149004623	gb|DQ022951.1	*TA*	Stress proteins
9	Rubisco activase	–	+	–	+	gi|34813618	gb|AF305876.2	*ZM*	Saccharide metabolism
10	PsbP (23 kDa extrinsic polypeptide of photosystem II)	+	–	+	–	gi|101383936	gb|AF052203.1|AF052203	*OS*	Photosynthetic ETC
11	Fructose-bisphosphate aldolase	+	+/−	++	+/−	gi|93014815	ref|NM_001036644.2	*AT*	Saccharide metabolism

AT  =  *Arabidopsis thaliana* (L.) Heynh.; ETC  =  electron transport chain; OEC  =  oxygen evolving complex of photosystem II; OS  =  *Oryza sativa* L.; LE  =  *Lycopersicon esculentum* Mill.; TA  =  *Triticum aestivum* L.; ZM  =  *Zea mays* L. The following symbols indicate the quantity of individual spots: –  =  absence, +/−  =  very weak intensity, +  =  medium intensity, ++  =  high intensity.

A comparison of the lists of proteins that were identified by the 2DGE approach and the iTRAQ technique showed only limited overlap in the outputs of these methods. Only 4 proteins among the 11 identified from the 2D gels were also found by the iTRAQ analysis: glutathione S-transferase GST27, Rubisco activase, 23-kDa extrinsic polypeptide of the photosystem II oxygen-evolving complex (PsbP) and the small heat-shock protein HSP26. Alterations in the levels of these proteins, as evaluated by the iTRAQ analysis, were found to be similar to the results from the 2DGE, supporting the credibility of these two methods. However, the 2DGE revealed 2 isoforms of HSP26 that responded to drought stress in different ways depending on the genotype (Figure 6), whereas both HSP26 isoforms identified by the iTRAQ were up-regulated by drought in both genotypes. However, the iTRAQ analysis revealed several other up- or down-regulated proteins that were present in various isoforms. The largest numbers of isoforms were observed for the HSP70 and 14-3-3 proteins (Table S1).

#### Differences between 2023 and CE704 genotypes

Based on the above-stated data and some additional observations, the two examined genotypes differed in several aspects of their morphology and physiology as well as in leaf proteome. Under conditions with sufficient water availability, CE704 was characterized by slightly lower plant height, smaller leaves and generally less DMS, as well as lower g_S_ and E compared with the 2023 genotype. Both genotypes did not significantly differ in their DMR, RWC, P_N_, c_i_ or activities of major antioxidant enzymes in leaves. When exposed to mild drought conditions at the early developmental stage, CE704 (in contrast to 2023) did not respond with early stomatal closure, maintained its original E and enhanced its P_N_; it also increased the amounts and activities of antioxidant enzymes. On the proteomic level, mild drought induced up-regulation of a much higher number of proteins in this genotype than in 2023 whereas the reverse was true for down-regulated proteins; this difference was particularly marked in the case of proteins involved in the regulation of translation. The drought-induced increase in level of various small heat shock proteins, chaperons, chaperonins and dehydrins was also usually much higher in the CE704 genotype compared with 2023.

## Discussion

### The Advantages of Maintaining the Stomata Open Under Mild Water Deficit

Plant tolerance to drought is often based either on escape by completing life cycle prior to the development of soil water deficit, or on dehydration avoidance using various strategies: maximum water acquisition through large root system, the ability to prevent water loss through decreased leaf growth, leaf senescence/shedding, leaf rolling, xeromorphic features of leaves or an early stomatal closure [1], [11], [26], [63]. The potential success of these individual strategies depends on many factors including drought severity, plant developmental stage, the concurrent action of another stressor (e.g. high irradiance or temperature) *etc.* Quite often, the trait that would be advantageous for plant under severe drought can have an opposite effect in the conditions of mild drought and *vice versa* [63]. For example, an early stomatal closure is usually regarded as a mechanism to avoid dehydration *via* reduction of transpiration and drought-tolerant maize genotypes have been previously described as having higher decrease of g_s_ and E induced by insufficient water supply [65]–[69].

The results of our study show that stomatal closure and significant decrease in the transpiration rate occurred even after mild drought conditions (6 d of gradual dehydration of the soil) in our sensitive genotype, 2023. Similar situation, *i.e.* an early closure of stomata and a rapid inhibition of photosynthetic CO_2_ assimilation was recently described for drought-sensitive cultivar of soybean [70]. In contrast, the more tolerant genotype CE704 did not display any such phenomenon and maintained open stomata and efficient transpiration. This condition probably led to a greater water loss from its leaves (as seen from its slightly higher decrease in the RWC values) but, at the same time, allowed for the maintenance of efficient photosynthesis. In fact, the sensitive genotype showed a slight (although statistically non-significant) decrease in P_N_ caused by drought, in contrast to CE704, which was characterized by highly efficient photosynthesis even after 6 days of drought (the values of P_N_ in the drought-stressed plants of this genotype actually increased compared with the control). Lopes *et al*. [11], as well as Tardieu [63] have suggested that genotypes displaying an early stomatal closure should have a good tolerance particularly under conditions of long and severe water deficit (*e.g.* in locations where plants experience terminal drought stress) because they would be able to decrease hydraulic gradients and to save soil water for a longer time than those with high g_s_. The drawback would be their lower growth capacity and potential biomass accumulation after the end of drought period, as the closure of stomata affects photosynthetic efficiency and, subsequently, biomass production. On the other hand, the more risky strategy of maintaining stomata open even under drought conditions would be beneficial under mild to moderate water deficits (such as in our case) or in conditions where periodical re-watering occurs, as the plant would be able to retain a relatively normal (possibly only slightly diminished) growth capacity. Thus, although low stomatal conductance is usually regarded as a general response of plants to drought conditions and as a trait associated with drought tolerance, it probably functions as such only under severe drought scenarios, whereas under mild water deficiencies, the maintenance of open stomata would be more profitable.

### The Response to Drought Stress is Characterized by the Up-regulation of Protective Proteins

In some cases, another strategy for drought tolerance can also play a role: the protection of cells from injury *via* various adjustments on biochemical and molecular level, particularly increased synthesis of various osmoprotectants and antioxidants, changes in cell wall elasticity, the induction of dehydrins and other proteins with a protective role (*e.g.* chaperones, repairing enzymes, proteins stabilizing thylakoid membranes) as well as specific stress-associated proteins involved in the regulation of transcription, post-transcriptional processes or signaling [1], [26], [60]. Drought stress can result in changes in the protein content through changes in gene expression or altered protein stability, degradation or modifications accompanying various cellular processes that reflect both drought-induced damage/metabolism failure and adjustment, adaptation and homeostasis maintenance. The majority of the proteins identified in our study responded to drought stress similarly (by an increase or a decrease in their levels) in both compared genotypes; however, approximately 38% of the differentially expressed proteins were up-regulated in one genotype and down-regulated in the other one. This finding indicates that the differential sensitivity of the examined genotypes to drought is associated with changes in a limited fraction of proteins and/or depends on the extent of the quantitative changes in protein levels. Similar results were observed by Peng *et al.*, who found cultivar-specific differences in the drought/salinity-induced changes (37% and 9% of differentially expressed proteins for the root and leaf proteomes, respectively) of the wheat proteome; many of these differences involved antioxidant proteins [36].

The most represented functional category of proteins responding to drought in our case contained various chaperones, chaperonins, heat-shock proteins and other proteins that participate in protein folding. These proteins were also among those that showed the strongest response to stress conditions, as identified by both iTRAQ and 2DGE analyses. All of these proteins were up-regulated during the stress period in both genotypes examined and the effect was more pronounced in the CE704 genotype. The strongest response was observed for several small heat shock proteins (sHSPs) that protect other proteins from denaturation and that facilitate the renaturation of misfolded proteins [71]. The accumulation of HSPs during dehydration is regarded as a general marker of plant stress tolerance and it has been observed that sHSPs accumulate to a large extent during drought and heat stress in the tolerant genotypes of wheat than in the sensitive ones [61]. Similarly, genotype-dependent changes in HSP levels (significantly correlated with carbon isotope discrimination) were observed in the leaves of eight poplar genotypes subjected to an insufficient water supply [72]. Xu and Huang reported an increase in the abundance of several HSPs in a drought-tolerant cultivar of Kentucky bluegrass but not in a drought-sensitive cultivar [57].

Dehydrins, the members of the second group of late embryogenesis abundant (LEA) proteins (9–200 kDa) [73], showed the greatest increase in their levels in our plants subjected to stress conditions, again particularly in the CE704 genotype. The expression of these hydrophilic, thermostable, glycine-rich proteins is known to be induced under dehydration in both tolerant and sensitive genotypes of various plant species [59], [74], [75]. These proteins accumulate simultaneously with other LEA proteins in response to different types of stress. Dehydrins are important for preserving the stability of membrane proteins and the adjustment of cell osmotic pressure as well as for macromolecular stabilization and the prevention of cell protein denaturation by the binding of water molecules to their surfaces [44]. Veeranagamallaiah *et al.* have also suggested that LEA proteins could act as a special form of molecular chaperones that would prevent the aggregation of other proteins induced by water stress [76].

Although proteins classified into the Detoxification category (*i.e.,* antioxidant enzymes) comprised only a small percentage of the proteins that were differentially expressed in the control and drought-stressed plants, a comparison of the changes in their levels with the changes in the activities of the respective antioxidant enzymes showed a similar, genotype-dependent trend. The levels of the proteins representing CAT, APX and SOD were up-regulated in the drought-tolerant CE704 genotype, which also showed an increase in the activities of these enzymes, whereas the drought-sensitive 2023 genotype was characterized by a down-regulation of CAT and APX and a lesser up-regulation of SOD compared with CE704, which again agreed with the biochemical data. The association between the levels/activities of antioxidant enzymes and plant drought tolerance has been previously observed, *e.g.,* in wheat [77]–[79], maize [80], rice [81], cowpea [82], [83], bean [84], [85] and poplar [86]. Proteomic studies performed in drought-tolerant and drought-sensitive cultivars of wheat [36], [87] and creeping bentgrass [58] showed that these cultivars differ with respect to the changes in the abundance of glutathione-S-transferase, CAT or APX, which, together with our results, clearly highlights the important role of these proteins in conveying tolerance to water stress. However, the amounts/activities of antioxidant enzymes cannot be generally employed as tolerance/sensitivity markers because the plant drought response is a much more complex process. It appears that the association between higher antioxidant capacity and drought tolerance is valid only under moderate stress, whereas under severe drought conditions, the overwhelming production of reactive oxygen species can no longer be balanced by the activity of antioxidant systems, even in genotypes/species that are drought-tolerant.

### The Sensitivity to Drought Stress Might be connected to the Differential Regulation of Proteosynthesis

The differential responses of the two examined genotypes to drought stress were also observed in proteins involved in the regulation of translation. The content of EF-TuM (a mitochondrial translation elongation factor) and a protein identified as subunit of eukaryotic transpation initiation factor 3 (eIF3), increased after 6 days of dehydration in the leaves of CE704 plants. However, in the drought-stressed plants of the sensitive genotype 2023, the amount of these proteins did not change much compared with the control plants. eIF3 is involved in initiation of proteosynthesis, it binds to the 40 S ribosome and, together with other initiation factors, promotes the binding of mRNA and methionyl-tRNA [88]. Simultaneously, the levels of the translation elongation factor eEF1D, which stimulates the exchange of GDP bound to EF-1α for GTP [89], were found to be strongly down-regulated in 2023 after a drought period but not in CE704 (which was characterized by high levels of this protein compared with 2023 even under control conditions). Similarly, Zhao *et al.* observed a decrease in the level of EF-Tu in a drought-sensitive bermudagrass genotype but not in a drought-tolerant genotype [59]. These changes in the levels of translation machinery components might be related to the down-regulated proteosynthesis in 2023, which is consistent with the generally lower number of up-regulated proteins found in this genotype after drought simulation and the generally lower level of their up-regulation.

An opposite trend observed for the accumulation of several ribosomal proteins (*i.e.,* an increase of their levels in the 2023 genotype) does not necessarily conflict with this view, as ribosomes are very stable cell structures and the observed increase in the amounts of certain ribosomal proteins in 2023 identified by the iTRAQ might be only relative with respect to the general decrease in the total protein content. Several recent studies on the response of the leaf proteome to drought stress also commented on the changes in the levels of ribosomal proteins; however, their observations differ. Tai *et al.* observed a strong down-regulation of ribosomal protein L28 in maize leaves subjected to moderate drought stress simulated by polyethylene glycol treatment [47]. Down-regulated levels of L21 ribosomal protein were found in the leaves of rice plants subjected to partial or whole root osmotic stress [41]. Similarly, Zhao *et al.* reported decreases in the levels of two ribosomal proteins (chloroplast ribosomal protein S1 and ribosomal protein L12) in a drought-sensitive genotype of bermudagrass; however, they also observed a significant increase in the level of another chloroplast ribosomal protein (S6) [59]. In contrast, the 40 S ribosomal protein SA (p40) was included in the group of significantly up-regulated proteins in a study conducted on the dehydrated leaves of the desiccation-tolerant grass *Sporobolus stapfianus* [90] and ribosomal protein L5 was observed to be up-regulated in two poplar species subjected to drought [91]. Clearly, the drought-induced regulation of proteosynthesis depends on the plant species and genotype and the length/severity of the simulated water-stress.

Drought-stressed plants of the CE704 genotype also exhibited higher level of several enzymes involved in amino acid metabolism, in contrast to the other genotype, which was characterized by the down-regulation of the majority of these proteins. This finding further supports our hypothesis regarding the differentially regulation of proteosynthesis in the two genotypes examined. Xu and Huang [57], who examined the response of the leaf proteome to drought in two cultivars of Kentucky bluegrass that differed in drought-tolerance, observed a somewhat similar situation, with lower decreases in the amounts of proteins associated with amino acid metabolism in the tolerant cultivar compared with the sensitive cultivar.

Several studies have demonstrated the impact of dehydration on the total leaf and root protein content. Yang *et al.* [91] described an increase in the total soluble protein content in the leaves of two poplar species subjected to an insufficient water supply; this increase was more efficient in the species that was better adapted to drought conditions. Mohammadkhani and Heidari [44] observed an increase in this parameter during an early phase of drought stress, which was probably caused by the expression of new stress-induced proteins. In contrast to this result, a prolonged period of drought caused a decline in the total protein content, but the extent of this decline depended on the intensity of the dehydration and on the length of the stress period. These authors speculated that such a decline could be caused by the intensified degradation of proteins and the limited availability of amino acids associated with the drought-induced inhibition of photosynthetic processes [44]. In our study, the increased content of proteases in the stressed plants of both genotypes also implies a higher rate of damaged/unnecessary protein degradation during stress conditions, indicating the need for the sensitive and selective regulation of both protein synthesis and degradation. Pinheiro *et al.* [92] have described a joint up-regulation of various proteases and protease inhibitors in drought-stressed lupin plants, suggesting a selective protein processing regulated by as-yet-unidentified mechanisms. Huerta-Ocampo *et al.* [51] reported an increased abundance of a member of the ubiquitin-conjugating enzymes family (participating in protein labeling for degradation in the proteasome system) in drought-stressed amaranth leaves and Aranjuelo *et al.* [52] noted an up-regulation of one of proteasome subunits in the leaves of alfalfa upon its subjection to a low water supply. The promotion of protein hydrolysis in maize leaves subjected to moderate drought stress was also observed by Tai *et al.* [47].

### The 2DGE and iTRAQ Analyses Provided Compatible but only Partially Overlapping Outcomes

The iTRAQ analysis is a second-generation proteomic technique that provides a gel-free shotgun quantitative analysis. This method allows the analysis of proteins that are represented in low quantities and those that tend to be difficult to separate by 2DGE. However, iTRAQ is a shotgun method that monitors several thousands of peptides without the possibility of the pre-selection of differentially represented peptides prior to mass spectrometry analysis. In contrast, the routinely used 2D gel methods (silver-stained or DIGE) allow the detection of lower numbers of protein spots, but subsequent mass spectrometry-based identification can be applied only on proteins that differ strongly among analyzed samples. In our study, we found only 17 such stress-regulated protein spots on silver-stained 2D gels, whereas the number of differentially expressed proteins detected by the iTRAQ (showing at least a twofold difference between any two samples) exceeded two hundred. The overlap between outputs of the two approaches was limited which results from different character of the two methods; both sample preparation and subsequent analysis differs significantly at many levels and supports identification of various peptides/proteins. Only relatively abundant proteins within a pI range of 3 to 11 can be detected by standard 2DGE, whereas iTRAQ method has completely different limitations and many peptides are not detected [93]. Therefore, a limited overlap in the outputs from gel-free and gel-based method is ordinary. Alvarez *et al.* [94] reported only 12% of proteins that were identified by both DIGE- and iTRAQ during their study of the root proteome in *Brassica juncea* plants exposed to cadmium.

Although both methods yielded relevant results when comparing between stressed and non-stressed plants of different genotypes, the 2DGE appears to be especially suitable for the detection of changes on the level of protein isoforms, as is clearly shown in the case of HSP26 protein, where the stress treatment resulted in an opposite regulation of different isoforms in the two genotypes. Different protein forms can be the products of paralogous genes or can originate from the same gene and differ by alternative splicing or posttranslational modifications. A 2DGE analysis of the total proteome of maize seeds found that only approximately 30% of the identified proteins were present as a single spot; most of the proteins were present in multiple isoforms, with as many as 26 spots [95]. Similarly, Vincent *et al.* [96] reported that more than 40% of the protein spots (out of 191 revealed by 2DGE) were redundant for drought-stressed grapevine. Although 2D gels cannot easily be replaced by another method for the identification of posttranslational modifications, gel-free approaches can also achieve the detection and quantification of protein paralogs if at least some paralog (isoform)-specific peptides are detected, as we have demonstrated for the proteins HSP70 and 14-3-3.

### How to Cope with Drought Stress: two Different Strategies Displayed by Tolerant and Sensitive Genotypes of Maize

Taking into account both the physiological responses of plants to drought stress and the changes in the leaf proteome, it is evident that the two genotypes compared in our study differ radically in their strategies for protecting themselves against dehydration-induced damage. The drought-stress conditions caused a rapid stomatal closure in the sensitive genotype 2023, leading to a reduction in its water loss from leaves but also to the inhibition of photosynthesis and proteosynthesis. In contrast, the significantly greater decrease in the RWC associated with the maintenance of open stomata in the tolerant genotype CE704 was accompanied by keeping these processes active. Understandably, it is difficult to uncover the natural causality in plant stress reactions, but our results lead us to speculate that the differences in the drought response of the analyzed maize genotypes might be connected primarily to their different sensitivities in stomatal closure under dehydration conditions and secondarily to the different biosynthesis of proteins participating in photosynthesis and/or protective pathways. The genotype CE704 appears to take a risk by keeping the stomata partially open even under drought conditions, which allows for the sufficient supply of CO_2_ and the maintenance (or even the strengthening) of active photosynthesis, enabling the synthesis of higher levels of various proteins/compounds that participate in cell protection/detoxification. The less profitable strategy of the sensitive genotype (at least under our experimental conditions of mild drought) probably results from its hypersensitive stomatal closure. This closure occurs when the water supply is already reduced and prevents further water loss but, at the same time, leads to a decrease in photosynthesis and the disabling of effective protective mechanisms that are dependent on the products of photosynthetic assimilation.

### Conclusions

Two alternative proteomic approaches, together with physiological analysis, were used to analyze the response to drought in two maize genotypes with different tolerances to dehydration. A comparison of the proteomic changes with the physiological parameters revealed completely different strategies for the two examined maize genotypes to cope with mild drought stress in the early developmental stage, which might be primarily connected to the sensitivity of stomatal closure during dehydration. Although the “classical” laborious gel-based method provided several unique results, the total number of identified proteins was substantially higher using the iTRAQ method. The output of the latter approach allowed the identification of many unique proteins with potential regulatory roles, thus providing a basis for the deeper understanding of drought stress response mechanisms.

## Materials and Methods

### Plant Material and Growth Conditions

Maize (*Zea mays* L.) plants of two inbred lines, the drought-tolerant CE704 and drought-sensitive 2023 genotypes, which originated from the breeding program of the *CEZEA Maize Breeding Station* (Čejč, Czech Republic) were used. These genotypes were selected based on their stress susceptibility (SSI; [97]) and stress tolerance (TOL; [98]) indices calculated from fresh or dry mass data collected from a larger genotypic set of 30 inbred lines; this analysis was made in the same conditions as the study presented here with stress intensity (SI) value 0.36 based on analysis of the fresh mass and 0.14 based on analysis of the dry mass of plant shoot. The values of SSI determined from the shoot fresh mass were 0.27 and 1.27, the values of TOL were 1.58 and 12.74 for CE704 and 2023, respectively. For the dry mass of whole plant, these values were −0.13 and 1.91 (SSI), −0.03 and 0.56 (TOL) for CE704 and 2023, respectively.

**Figure 7:**
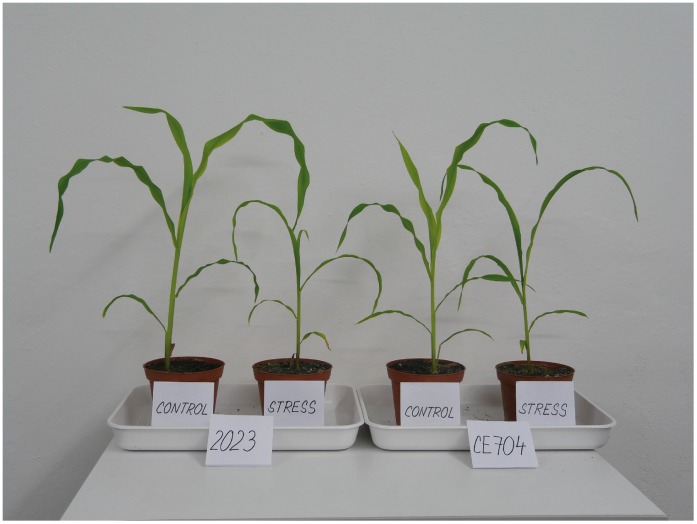
Phenotypic representation of drought-stressed and control plants of two maize genotypes. 2023: sensitive genotype; CE704: tolerant genotype.

Kernels were sown into pots (12 cm diameter, 13 cm depth, one kernel per pot) that were filled with a mixture of garden soil and sand (2∶1 v/v), placed in a naturally lit greenhouse under semi-controlled conditions (air temperature 25/20°C and relative air humidity 50/60% day/night) and sufficiently watered with tap water. At 34 days from the date of sowing (at this time, all plants of both genotypes had 3–4 fully developed leaves), one half of the plants continued to be normally watered (control), whereas the second half of the plants (stressed) was subjected to mild drought simulation by withholding the water for 6 days. At the end of this period, the volumetric soil water content (measured at the 5 cm depth from the top of soil level with *WET-2 Sensor/HH2 Moisture Meter, Delta-T Devices*, Cambridge, Great Britain) was approximately 12.5% for the stressed plants, compared with approximately 30% for the control plants. No differences between both genotypes in the volumetric soil water content were found. Phenotypic representation of both control and stressed plants of both genotypes is shown in Figure 7. The experiments were conducted in two independent series with a completely randomized design; each variant (genotype/water treatment combination) in each series was represented by 60 plants, which were utilized for morphological measurements and leaf relative water content (RWC) determination, gas exchange measurements, analyses of the activities of antioxidant enzymes and the sampling necessary for the proteomic analyses.

### Morphological Measurements and Determination of Leaf Relative Water Content

The height of the plants (measured from the ground to the ligule of the youngest fully developed leaf) and the dry masses of the shoot (DMS) and roots (DMR) after drying at 80°C for at least 5 days were recorded at the end of the drought simulation period. The RWC was established as 100×(FM–DM)/(SM–DM), where FM represents the fresh mass of 10 leaf discs (diameter 6 mm) cut from the middle portion of the 4^th^ leaf blade and immediately weighed on analytical balances with 0.1 mg precision, SM is the saturated mass of the same discs after their hydration in the dark for 5 h and DM is the dry mass of these discs after they were oven-dried at 80°C for 48 h. The specific leaf weight (SLW) was determined from the same discs. Each genotype/water treatment combination was analyzed in 18 replicates representing independent plants.

### Leaf Gas Exchange Measurements

The middle portion of the 4^th^ leaf was used for the measurements of the net photosynthetic rate (P_N_)_,_ the rate of transpiration (E), the stomatal conductance (g_S_) and the intercellular CO_2_ concentration (c_i_). These parameters were measured on the leaves *in situ* using the portable gas exchange system *LCpro+* (*ADC BioScientific Ltd.*, Hoddesdon, Great Britain) between 8∶00 and 11∶00, Central European time. The irradiance was 650 µmol m^−2^ s^−1^ of photosynthetically active radiation, the temperature in the measurement chamber was 25°C, the CO_2_ concentration was 550±50 µL L^−1^, the air flow rate was 205±30 µmol s^−1^ and the duration of the measurement of each sample was 10 min after the establishment of steady-state conditions inside the measurement chamber. The water use efficiency (WUE) was calculated as the ratio of P_N_/E. Each parameter was measured in 20–22 independent replicates (individual plants) per variant.

### Determination of the Activities of Antioxidant Enzymes

Soluble protein extracts were prepared by leaf homogenization (the sampling was performed from 10∶30 to 11∶00, Central European time) in 0.1 M Tris-HCl extraction buffer containing 1 mM dithiothreitol (DTT), 1 mM 2,2′,2′′,2′′′-(Ethane-1,2-diyldinitrilo)tetraacetic acid (EDTA), 1 % (w:v) Triton X-100 and 5 mM ascorbic acid (pH 7.8) at a ratio of 5 cm^3^ per g fresh weight, as described by Holá *et al.* [99]. The samples were frozen in liquid nitrogen and stored at –70°C until the determination of the activities of the antioxidant enzymes.

The activities of ascorbate peroxidase (APX, EC 1.11.1.11), glutathione reductase (GR, EC 1.6.4.2) and superoxide dismutase (SOD, EC 1.15.1.1) were measured spectrophotometrically (*Hitachi U 3300*, *Hitachi High-Tech Corporation*, Tokyo, Japan) at 25°C. The activity of APX was determined by the decrease in reduced ascorbate at 290 nm, as described by Nakano and Asada [100]. The GR activity was assayed, as described by Smith *et al.* [101], by the increase in absorbance at 412 nm due to the formation of a colored complex of reduced glutathione, produced by GR, with 5-(3-carboxy-4-nitrophenyl)disulfanyl-2-nitrobenzoic acid (DTNB). The SOD activity was measured at 470 nm; the production of superoxide was provided by the conversion of xanthine catalyzed by xanthine oxidase [102]. One unit of SOD activity was defined as the amount of the enzyme required for 50% inhibition of the reaction rate of 2,3-bis(2-methoxy-4-nitro-5-sulfophenyl)-2H-tetrazolium-5-carboxanilide inner salt (XTT), a detection molecule reduced by superoxide. The activity of catalase (CAT, EC 1.11.1.6) was measured polarographically using an oxygen electrode (*Hansatech Instruments*, Great Britain), as described by Thomas *et al.* [103]. The protein contents were determined spectrophotometrically by the Bradford assay [104] using bovine serum albumin as a standard. The total number of plants per experimental variant used for the preparation of the necessary mixture samples was 50–60, which provided eight replications for the statistical analysis.

### Preparation of Protein Extracts, Two-dimensional Gel Electrophoresis and iTRAQ Analysis

Samples produced from a mixture of the 4^th^ leaves of approximately 10 plants per genotype/water treatment combination were used for the proteomic analyses. The total protein extraction procedure was performed as described by Görg *et al.* [105]. The leaf tissue was homogenized in liquid nitrogen and extracted with 20% trichloroacetic acid in acetone.

For the 2DGE analysis, the total proteins were extracted from the dried precipitates with a lysis solution containing urea, 3-[(3-cholamidopropyl)dimethylammonio]-1-propanesulfonate (CHAPS) and DTT [105]. The isoelectric focusing was performed according to the manufacturer’s instructions on pre-made polyacrylamide gel strips with immobilized pH gradients (*ReadyStrip™, BioRad*, Hercules, CA, U.S.A.). Strips with a broad pH range (3–10) were used for the initial experiments to compare the broad spectrum of proteins. For more detailed comparisons, strips with a narrower pH range (4–7) were used. Sodium dodecylsulfate-polyacrylamide gel electrophoresis was used to separate proteins in the second dimension, after which the gels were stained with silver [106]. Silver-stained gels displaying the leaf proteome of the control and drought-stressed plants of both examined genotypes were manually compared in all of the relevant combinations, *i.e.,* between genotypes subjected to the same treatment or between control and stressed plants of the same genotype. At least three independent replicates were generated for each type of gel to verify the observed differences. The spots that differed in their presence or intensity between variants were isolated and analyzed by MALDI-TOF (matrix assisted laser desorption/ionization – time of flight) mass spectrometry (MS) after trypsin cleavage.

For the iTRAQ (isobaric tag for relative and absolute quantitation) analysis, dried precipitates (100 µg each) were dissolved in buffer provided in iTRAQ reagent kit (*AB Sciex*, Framingham, U.S.A.) and treated as described by the manufacturer. The labeled samples were pooled and precipitated with acetone. The pellet was dissolved in 2 M urea in LC-MS grade water prior to isoelectric focusing on 7-cm immobilized pH gradient strips (pH 3–10) (*BioRad*) for 20,000 VHrs. The strip was cut into 26 slices, which were separately extracted with 50% acetonitrile and 1% trifluoroacetic acid. The supernatant was diluted 1∶1 with water and used for LC-MALDI.

The LC-MALDI analyses were performed on an *Ultimate 3000 HPLC system* (*Dionex*, Sunnyvale, U.S.A.) that was coupled to a *Probot* micro-fraction collector (*Dionex*). Tryptic peptides were loaded onto a *PepMap 100 C18 RP* column (3-µm particle size, 15-cm length, 75-µm internal diameter; *Dionex*) and separated by a gradient of 5% (v/v) acetonitrile (ACN), 0.1% (v/v) trifluoroacetic acid (TFA) to 80% (v/v) ACN, 0.1% (v/v) TFA over a period of 60 min. The flow rate was set to 300 nL/min. The eluate was mixed 1∶3 with matrix solution (2 mg/mL α-cyano-4-hydroxycinnamic acid in 80% ACN) prior to spotting onto a MALDI target. The spectra were acquired on a *4800 Plus MALDI TOF/TOF analyzer* (*AB Sciex*) equipped with a *Nd:YAG* laser (355 nm, firing rate 200 Hz). First, all of the spots were measured in MS mode and then, up to 12 of the strongest precursors were selected for MS/MS analysis, which was performed with 1 kV of collision energy and an operating pressure of collision cell set to 10^−6^ Torr.

The peak lists from the MS/MS spectra were generated using *GPS Explorer v. 3.6* (*AB Sciex*) and searched by locally installed *Mascot v. 2.1* (*Matrix Science*) against the NCBInr protein database (*i.e.,* all non-redundant GenBank CDS translations + PDB + SwissProt + PIR + PRF) and a database of expressed sequence tags (EST) downloaded from GenBank. The database search criteria were as follows – enzyme: trypsin; taxonomy: *Zea mays*; fixed modifications: S-methyl methanethiosulfonate modification of cysteines, iTRAQ on N-terminus and ε-amino group of lysine; variable modification: methionine oxidation; peptide mass tolerance: 120 ppm, allowed one missed cleavage site; MS/MS tolerance: 0.2 Da; maximum peptide rank: 1; minimum ion score C.I. (peptide): 95%. The quantification was performed by the *GPS Explorer* software *v. 3.6* (*AB Sciex*) and the ratios for the individual proteins were normalized in *GPS Explorer*.

### Statistical Analysis

The data were subjected to one-way analysis of variance followed by Tukey-Kramer tests (with a probability level of 0.05 treated as statistically significant) for the comparisons between individual genotype/water treatment combinations. The *CoStat* computer program, *version 6.204* (*CoHort Software*, Monterey, CA, USA) was used for all statistical evaluations.

## Supporting Information

Table S1 The list of identified proteins/ESTs and their classification to functional categories. Sheets “Proteins NCBInr” and “ESTs” show details to identification and quantification of all matching iTRAQ-labelled peptides characterized by tandem MS/MS. Yellow-labelled columns in these sheets show different ratios between genotypes 2023 and CE704 and/or cultivation conditions (C, control; S, stress). Blue-labelled collumns show the same ratios; however, in case the respective ratio was lower than 1, these ratios were expressed as –1/the respective ratio. Green-labelled column shows derived ratio reflecting difference in the response of both genotypes to drought stress. Functional categories sheets present only proteins, in which the absolute values of any of the following ratios (or –1/the respective ratio) exceeded 2: 2023S/2023C and CE704S/CE704C ratios reflecting stress-induced responses in individual genotypes, the derived ratio (CE704S/CE704C)/(2023S/2023C) reflecting difference in the response of both genotypes to drought stress.
(XLS)

## References

[1] Farooq M, Wahid A, Kobayashi N, Fujita D, Basra SMA (2009). Plant drought stress: effects, mechanisms and management.. Agron Sustain Dev.

[2] Pinheiro C, Chaves MM (2011). Photosynthesis and drought: can we make metabolic connections from available data?. J Exp Bot.

[3] Chernyad’ev II (2005). Effect of water stress on the photosynthetic apparatus of plants and the protective role of cytokinins: A review.. Appl Biochem Microbiol.

[4] Medrano H, Escalona JM, Bota J, Gulias J, Flexas J (2002). Regulation of photosynthesis of C-3 plants in response to progressive drought: Stomatal conductance as a reference parameter.. Ann Bot.

[5] Pospisilova J, Vagner M, Malbeck J, Travniakova A, Batkova P (2005). Interactions between abscisic acid and cytokinins during water stress and subsequent rehydration.. Biol Plant.

[6] Seki M, Umezawa T, Kim JM, Matsui A, To T, Jenks MA, Hasegawa PM, Jain SM (2007). Transcriptome analysis of plant drought and salt stress response..

[7] Wilkinson S, Davies WJ (2002). ABA-based chemical signalling: the co-ordination of responses to stress in plants.. Plant Cell Environ.

[8] Wilkinson S, Davies WJ (2010). Drought, ozone, ABA and ethylene: new insights from cell to plant to community.. Plant Cell Environ.

[9] Chaves MM, Flexas J, Pinheiro C (2009). Photosynthesis under drought and salt stress: regulation mechanisms from whole plant to cell.. Ann Bot.

[10] Lawlor DW, Tezara W (2009). Causes of decreased photosynthetic rate and metabolic capacity in water-deficient leaf cells: a critical evaluation of mechanisms and integration of processes.. Ann Bot.

[11] Lopes MS, Araus JL, van Heerden PDR, Foyer CH (2011). Enhancing drought tolerance in C(4) crops.. J Exp Bot.

[12] Chaves MM, Maroco JP, Pereira JS (2003). Understanding plant responses to drought - from genes to the whole plant.. Funct Plant Biol.

[13] Flexas J, Medrano H (2002). Drought-inhibition of photosynthesis in C-3 plants: Stomatal and non-stomatal limitations revisited.. Ann Bot.

[14] Flexas J, Bota J, Loreto F, Cornic G, Sharkey TD (2004). Diffusive and metabolic limitations to photosynthesis under drought and salinity in C(3) plants.. Plant Biol.

[15] Lawlor DW (2002). Limitation to photosynthesis in water-stressed leaves: Stomata vs. metabolism and the role of ATP.. Ann Bot.

[16] Yordanov I, Velikova V, Tsonev T (2003). Plant responses to drought and stress tolerance.. Bulg J Plant Physiol 187–206.

[17] Parry MAJ, Andralojc PJ, Khan S, Lea PJ, Keys AJ (2002). Rubisco activity: Effects of drought stress.. Ann Bot.

[18] Reddy AR, Chaitanya KV, Vivekanandan M (2004). Drought-induced responses of photosynthesis and antioxidant metabolism in higher plants.. J Plant Physiol.

[19] Lawlor DW, Cornic G (2002). Photosynthetic carbon assimilation and associated metabolism in relation to water deficits in higher plants.. Plant Cell Environ.

[20] Ghannoum O (2009). C(4) photosynthesis and water stress.. Ann Bot.

[21] Cornic G, Fresneau C (2002). Photosynthetic carbon reduction and carbon oxidation cycles are the main electron sinks for photosystem II activity during a mild drought.. Ann Bot.

[22] Chaves MM, Pereira JS, Maroco J, Rodrigues ML, Ricardo CPP (2002). How plants cope with water stress in the field. Photosynthesis and growth.. Ann Bot.

[23] Langridge P, Paltridge N, Fincher G (2006). Functional genomics of abiotic stress tolerance in cereals.. Brief Funct Genomics Proteomics.

[24] Valliyodan B, Nguyen HT (2006). Understanding regulatory networks and engineering for enhanced drought tolerance in plants.. Curr Opin Plant Biol.

[25] Verbruggen N, Hermans C (2008). Proline accumulation in plants: a review.. Amino Acids.

[26] Bray EA, Jenks MA, Hasegawa PM, Jain SM (2007). Molecular and physiological responses to water-deficit stress..

[27] Cohen D, Bogeat-Triboulot MB, Tisserant E, Balzergue S, Martin-Magniette ML (2010). Comparative transcriptomics of drought responses in Populus: a meta-analysis of genome-wide expression profiling in mature leaves and root apices across two genotypes.. BMC Genomics 11.

[28] Gong PJ, Zhang JH, Li HX, Yang CX, Zhang CJ (2010). Transcriptional profiles of drought-responsive genes in modulating transcription signal transduction, and biochemical pathways in tomato.. J Exp Bot.

[29] Hayano-Kanashiro C, Calderon-Vazquez C, Ibarra-Laclette E, Herrera-Estrella L, Simpson J (2009). Analysis of Gene Expression and Physiological Responses in Three Mexican Maize Landraces under Drought Stress and Recovery Irrigation.. PLoS ONE 4.

[30] Matsui A, Ishida J, Morosawa T, Mochizuki Y, Kaminuma E (2008). Arabidopsis transcriptome analysis under drought, cold, high-salinity and ABA treatment conditions using a tiling array.. Plant Cell Physiol.

[31] Seki M, Narusaka M, Ishida J, Nanjo T, Fujita M (2002). Monitoring the expression profiles of 7000 Arabidopsis genes under drought, cold and high-salinity stresses using a full-length cDNA microarray.. Plant J.

[32] Shinozaki K, Yamaguchi-Shinozaki K (2007). Gene networks involved in drought stress response and tolerance.. J Exp Bot.

[33] Zheng J, Zhao JF, Tao YZ, Wang JH, Liu YJ (2004). Isolation and analysis of water stress induced genes in maize seedlings by subtractive PCR and cDNA macroarray.. Plant Mol Biol.

[34] Bogeat-Triboulot MB, Brosche M, Renaut J, Jouve L, Le Thiec D (2007). Gradual soil water depletion results in reversible changes of gene expression, protein profiles, ecophysiology, and growth performance in Populus euphratica, a poplar growing in arid regions.. Plant Physiol.

[35] Kottapalli KR, Rakwal R, Shibato J, Burow G, Tissue D (2009). Physiology and proteomics of the water-deficit stress response in three contrasting peanut genotypes.. Plant Cell Environ.

[36] Peng ZY, Wang MC, Li F, Lu HJ, Li CL (2009). A Proteomic Study of the Response to Salinity and Drought Stress in an Introgression Strain of Bread Wheat.. Mol Cell Proteom.

[37] Ali GM, Komatsu S (2006). Proteomic analysis of rice leaf sheath during drought stress.. J Proteome Res.

[38] Ke YQ, Han GQ, He HQ, Li JX (2009). Differential regulation of proteins and phosphoproteins in rice under drought stress.. Biochem Biophys Res Commun.

[39] Salekdeh GH, Siopongco J, Wade LJ, Ghareyazie B, Bennett J (2002). Proteomic analysis of rice leaves during drought stress and recovery.. Proteomics.

[40] Salekdeh GH, Siopongco J, Wade LJ, Ghareyazie B, Bennett J (2002). A proteomic approach to analyzing drought- and salt-responsiveness in rice.. Field Crops Res.

[41] Shu LB, Ding W, Wu JH, Feng FJ, Luo LJ (2010). Proteomic Analysis of Rice Leaves Shows the Different Regulations to Osmotic Stress and Stress Signals.. J Integr Plant Biol.

[42] Xiong JH, Fu BY, Xu HX, Li YS (2010). Proteomic analysis of PEG-simulated drought stress-responsive proteins of rice leaves using a pyramiding rice line at the seedling stage.. Bot Stud.

[43] de Vienne D, Leonardi A, Damerval C, Zivy M (1999). Genetics of proteome variation for QTL characterization: application to drought-stress responses in maize.. J Exp Bot.

[44] Mohammadkhani N, Heidari R (2008). Effects of drought stress on soluble proteins in two maize varieties.. Turk J Biol.

[45] Riccardi F, Gazeau P, de Vienne D, Zivy M (1998). Protein changes in response to progressive water deficit in maize - Quantitative variation and polypeptide identification.. Plant Physiol.

[46] Riccardi F, Gazeau P, Jacquemot MP, Vincent D, Zivy M (2004). Deciphering genetic variations of proteome responses to water deficit in maize leaves.. Plant Physiol Biochem.

[47] Tai FJ, Yuan ZL, Wu XL, Zhao PF, Hu XL (2011). Identification of membrane proteins in maize leaves, altered in expression under drought stress through polyethylene glycol treatment.. Plant Omics.

[48] Vincent D, Lapierre C, Pollet B, Cornic G, Negroni L (2005). Water deficits affect caffeate O-methyltransferase, lignification, and related enzymes in maize leaves. A proteomic investigation.. Plant Physiol.

[49] Caruso G, Cavaliere C, Foglia P, Gubbiotti R, Samperi R (2009). Analysis of drought responsive proteins in wheat (Triticum durum) by 2D-PAGE and MALDI-TOF mass spectrometry.. Plant Sci.

[50] Parida AK, Dagaonkar VS, Phalak MS, Umalkar GV, Aurangabadkar LP (2007). Alterations in photosynthetic pigments, protein and osmotic components in cotton genotypes subjected to short-term drought stress followed by recovery.. Plant Biotechnol Rep.

[51] Huerta-Ocampo JA, Briones-Cerecero EP, Mendoza-Hernandez G, De Leon-Rodriguez A, de la Rosa APB (2009). Proteomic Analysis of Amaranth (Amaranthus Hypochondriacus L.) Leaves Under Drought Stress.. Int J Plant Sci.

[52] Aranjuelo I, Molero G, Erice G, Avice JC, Nogues S (2011). Plant physiology and proteomics reveals the leaf response to drought in alfalfa (Medicago sativa L.).. J Exp Bot.

[53] Hajheidari M, Abdollahian-Noghabi M, Askari H, Heidari M, Sadeghian SY (2005). Proteome analysis of sugar beet leaves under drought stress.. Proteomics.

[54] Fulda S, Mikkat S, Stegmann H, Horn R (2011). Physiology and proteomics of drought stress acclimation in sunflower (Helianthus annuus L.).. Plant Biol.

[55] Bonhomme L, Monclus R, Vincent D, Carpin S, Claverol S (2009). Genetic variation and drought response in two Populus x euramericana genotypes through 2-DE proteomic analysis of leaves from field and glasshouse cultivated plants.. Phytochemistry.

[56] Xiao XW, Yang F, Zhang S, Korpelainen H, Li CY (2009). Physiological and proteomic responses of two contrasting Populus cathayana populations to drought stress.. Physiol Plant.

[57] Xu CP, Huang BR (2010). Comparative Analysis of Drought Responsive Proteins in Kentucky Bluegrass Cultivars Contrasting in Drought Tolerance.. Crop Sci.

[58] Xu CP, Huang BR (2010). Differential proteomic responses to water stress induced by PEG in two creeping bentgrass cultivars differing in stress tolerance.. J Plant Physiol.

[59] Zhao Y, Du HM, Wang ZL, Huang BR (2011). Identification of proteins associated with water-deficit tolerance in C(4) perennial grass species, Cynodon dactylon x Cynodon transvaalensis and Cynodon dactylon.. Physiol Plant.

[60] Ashraf M (2010). Inducing drought tolerance in plants: Recent advances.. Biotechnol Adv.

[61] Salekdeh GH, Komatsu S (2007). Crop proteomics: Aim at sustainable agriculture of tomorrow.. Proteomics.

[62] Shao HB, Chu LY, Jaleel CA, Manivannan P, Panneerselvam R (2009). Understanding water deficit stress-induced changes in the basic metabolism of higher plants - biotechnologically and sustainably improving agriculture and the ecoenvironment in arid regions of the globe.. Crit Rev Biotechnol.

[63] Tardieu F (2012). Any trait or trait-related allele can confer drought tolerance: just design the right drought scenario.. J Exp Bot.

[64] Ribaut JM, Betran J, Monneveux P, Setter T, Bennetzen JL, Hake SC (2012). Drought tolerance in maize..

[65] Cattivelli L, Rizza F, Badeck FW, Mazzucotelli E, Mastrangelo AM (2008). Drought tolerance improvement in crop plants: An integrated view from breeding to genomics.. Field Crops Res.

[66] Grzesiak MT, Grzesiak S, Skoczowski A (2006). Changes of leaf water potential and gas exchange during and after drought in triticale and maize genotypes differing in drought tolerance.. Photosynthetica.

[67] Grzesiak MT, Rzepka A, Hura T, Hura K, Skoczowski A (2007). Changes in response to drought stress of triticale and maize genotypes differing in drought tolerance.. Photosynthetica.

[68] Kumari M, Dass S, Vimala Y, Arora P (2004). Physiological parameters governing drought tolerance in maize.. Indian J Plant Physiol.

[69] Zarco-Perelló E, Gonzáles-Hernández VA, López-Peralta MC, Sallinas-Moreno Y (2005). Physiological markers for drought tolerance in maize (*Zea mays* L.).. Agrociencia.

[70] Fenta BA, Driscoll SP, Kunert KJ, Foyer CH (2012). Characterization of drought-tolerance traits in nodulated soya beans: The importance of maintaining photosynthesis and shoot biomass under drought-induced limitations on nitrogen metabolism.. J Agron Crop Sci.

[71] Taylor NL, Tan YF, Jacoby RP, Millar AH (2009). Abiotic environmental stress induced changes in the Arabidopsis thaliana chloroplast, mitochondria and peroxisome proteomes.. J Proteomics.

[72] Bonhomme L, Monclus R, Vincent D, Carpin S, Lomenech AM (2009). Leaf proteome analysis of eight Populus xeuramericana genotypes: Genetic variation in drought response and in water-use efficiency involves photosynthesis-related proteins.. Proteomics.

[73] Close TJ (1996). Dehydrins: Emergence of a biochemical role of a family of plant dehydration proteins.. Physiol Plant.

[74] Cellier F, Conejero G, Breitler JC, Casse F (1998). Molecular and physiological responses to water deficit in drought-tolerant and drought-sensitive lines of sunflower - Accumulation of dehydrin transcripts correlates with tolerance.. Plant Physiol.

[75] Wood AJ, Goldsbrough PB (1997). Characterization and expression of dehydrins in water-stressed Sorghum bicolor.. Physiol Plant.

[76] Veeranagamallaiah G, Prasanthi J, Reddy KE, Pandurangaiah M, Babu OS (2011). Group 1 and 2 LEA protein expression correlates with a decrease in water stress induced protein aggregation in horsegram during germination and seedling growth.. J Plant Physiol.

[77] Lascano HR, Antonicelli GE, Luna CM, Melchiorre MN, Gomez LD (2001). Antioxidant system response of different wheat cultivars under drought: field and in vitro studies.. Austr J Plant Physiol.

[78] Loggini B, Scartazza A, Brugnoli E, Navari-Izzo F (1999). Antioxidative defense system, pigment composition, and photosynthetic efficiency in two wheat cultivars subjected to drought.. Plant Physiol.

[79] Sairam RK, Saxena DC (2000). Oxidative stress and antioxidants in wheat genotypes: Possible mechanism of water stress tolerance.. J Agron Crop Sci.

[80] Pastori GM, Trippi VS (1992). Oxidative Stress Induces High-Rate of Glutathione-Reductase Synthesis in A Drought-Resistant Maize Strain.. Plant Cell Physiol.

[81] Guo Z, Ou W, Lu S, Zhong Q (2006). Differential responses of antioxidative system to chilling and drought in four rice cultivars differing in sensitivity.. Plant Physiol Biochem.

[82] Arcy-Lameta A, Ferrari-Iliou R, Contour-Ansel D, Pham-Thi AT, Zuily-Fodil Y (2006). Isolation and characterization of four ascorbate peroxidase cDNAs responsive to water deficit in cowpea leaves.. Ann Bot.

[83] Contour-Ansel D, Torres-Franklin ML, De Carvalho MHC, Arcy-Lameta A (2006). Glutathione reductase in leaves of cowpea: Cloning of two cDNAs, expression and enzymatic activity under progressive drought stress, desiccation and abscisic acid treatment.. Ann Bot.

[84] Torres-Franklin ML, Contour-Ansel D, Zuily-Fodil Y, Pham-Thi AT (2008). Molecular cloning of glutathione reductase cDNAs and analysis of GR gene expression in cowpea and common bean leaves during recovery from moderate drought stress.. J Plant Physiol.

[85] Turkan I, Bor M, Ozdemir F, Koca H (2005). Differential responses of lipid peroxidation and antioxidants in the leaves of drought-tolerant P-acutifolius Gray and drought-sensitive P-vulgaris L. subjected to polyethylene glycol mediated water stress.. Plant Sci.

[86] Edjolo A, Laffray D, Guerrier G (2001). The ascorbate-glutathione cycle in the cytosolic and chloroplastic fractions of drought-tolerant and drought-sensitive poplars.. J Plant Physiol.

[87] Hajheidari M, Eivazi A, Buchanan BB, Wong JH, Majidi I (2007). Proteomics uncovers a role for redox in drought tolerance in wheat.. J Proteome Res.

[88] Trachsel H, Staehelin T (1979). Initiation of Mammalian Protein-Synthesis - Multiple Functions of the Initiation-Factor Eif-3.. Biochim Biophys Acta.

[89] Peters HI, Chang YWE, Traugh JA (1995). Phosphorylation of Elongation-Factor 1(Ef-1) by Protein-Kinase-C Stimulates Gdp/Gtp-Exchange Activity.. Eur J Biochem.

[90] Oliver MJ, Jain R, Balbuena TS, Agrawal G, Gasulla F (2011). Proteome analysis of leaves of the desiccation-tolerant grass, Sporobolus stapfianus, in response to dehydration.. Phytochemistry.

[91] Yang F, Wang Y, Miao LF (2010). Comparative physiological and proteomic responses to drought stress in two poplar species originating from different altitudes.. Physiol Plant.

[92] Pinheiro C, Kehr J, Ricardo CP (2005). Effect of water stress on lupin stem protein analysed by two-dimensional gel electrophoresis.. Planta.

[93] Wu WW, Wang GH, Baek SJ, Shen RF (2006). Comparative study of three proteomic quantitative methods, DIGE, cICAT, and iTRAQ, using 2D gel- or LC-MALDI TOF/TOF.. J Proteome Res.

[94] Alvarez S, Berla BM, Sheffield J, Cahoon RE, Jez JM (2009). Comprehensive analysis of the Brassica juncea root proteome in response to cadmium exposure by complementary proteomic approaches.. Proteomics.

[95] Mechin V, Balliau T, Chateau-Joubert S, Davanture M, Langella O (2004). A two-dimensional proteome map of maize endosperm.. Phytochemistry.

[96] Vincent D, Ergul A, Bohlman MC, Tattersall EAR, Tillett RL (2007). Proteomic analysis reveals differences between Vitis vinifera L. cv. Chardonnay and cv. Cabernet Sauvignon and their responses to water deficit and salinity.. J Exp Bot.

[97] Fischer RA, Maurer R (1978). Drought resistance in spring wheat cultivars. I. Grain yield responses.. Austr J Agric Res.

[98] Rosielle AA, Hamblin J (1981). Theoretical aspects of selection for yield in stress and non-stress environments.. Crop Sci.

[99] Hola D, Kocova M, Rothova O, Wilhelmova N, Benesova M (2007). Recovery of maize (Zea mays L.) inbreds and hybrids from chilling stress of various duration: Photosynthesis and antioxidant enzymes.. J Plant Physiol.

[100] Nakano Y, Asada K (1981). Hydrogen-Peroxide Is Scavenged by Ascorbate-Specific Peroxidase in Spinach-Chloroplasts.. Plant Cell Physiol.

[101] Smith IK, Vierheller TL, Thorne CA (1988). Assay of Glutathione-Reductase in Crude Tissue-Homogenates Using 5,5′-Dithiobis(2-Nitrobenzoic Acid).. Anal Biochem.

[102] Ukeda H, Maeda S, Ishii T, Sawamura M (1997). Spectrophotometric assay for superoxide dismutase based on tetrazolium salt 3′-{1-[(phenylamino)-carbonyl]-3,4-tetrazolium}-bis(4-methoxy-6-nitro)benzenesulfonic acid hydrate reduction by xanthine-xanthine oxidase.. Anal Biochem.

[103] Thomas DJ, Avenson TJ, Thomas JB, Herbert SK (1998). A cyanobacterium lacking iron superoxide dismutase is sensitized to oxidative stress induced with methyl viologen but is not sensitized to oxidative stress induced with norflurazon.. Plant Physiol.

[104] Bradford MM (1976). Rapid and Sensitive Method for Quantitation of Microgram Quantities of Protein Utilizing Principle of Protein-Dye Binding.. Anal Biochem.

[105] Gorg A, Obermaier C, Boguth G, Harder A, Scheibe B (2000). The current state of two-dimensional electrophoresis with immobilized pH gradients.. Electrophoresis.

[106] Blum H, Beier H, Gross HJ (1987). Improved Silver Staining of Plant-Proteins, Rna and Dna in Polyacrylamide Gels.. Electrophoresis.

